# Assessing the determinants of teachers’ job happiness in the private universities

**DOI:** 10.3389/fpsyg.2022.1018517

**Published:** 2022-12-12

**Authors:** Baohua Chen, Guangxin Ren, Yanjun Liu

**Affiliations:** ^1^President’s Office, Dongguan City University, Dongguan, China; ^2^Department of Education and Teaching, Northern Investment Group, Beijing, China; ^3^Institute of Higher Education, Anhui University, Hefei, China

**Keywords:** professional identity, job competence, professional motivation, professional prospects, perceived fairness, job achievements, job happiness

## Abstract

Teachers’ job happiness in private universities is an important element for the healthy and orderly development of universities and an inevitable requirement for the construction of university faculty, and it has become a hot topic of research in the field of private higher education at present. However, there is still a lack of empirical studies on the factors influencing job happiness in private universities. This study constructs a theoretical model between professional identity, job competence, professional motivation, professional prospects, perceived fairness, job achievements and job happiness, and explores the specific drivers of teachers’ job happiness in private universities based on empirical research. The results of the data analysis showed that professional identity, job competence, professional prospects, perceived fairness, job achievements, and professional motivation all had significant effects on teachers’ job happiness, and the effects were decreasing. This study examined the effects of job happiness in practice in private universities, which helped private universities to enhance teachers’ professional identity, strengthen organizational support for teacher development, promote teachers’ teaching ability, improve job competence, and build a developmental teacher evaluation mechanism.

## Introduction

Private universities in China have experienced nearly three decades of development, from scratch, from the pursuit of scale to connotative development, all of which have made important contributions to the popularization and high-quality development of higher education in China. However, compared with public higher education, private universities face bottlenecks to high-quality development, such as weak national policy support, internal teaching, research and student management models still need to be upgraded, teachers’ salaries and social welfare levels are low, infrastructure needs to be consolidated and updated, and faculty members are unstable and turnover is frequent (Xu and Zhang, 2017). With the development of education, teachers are becoming more and more specialized, and their job happiness is not optimistic. The job happiness of teachers in private universities is a serious challenge, and there are many differences between private universities and public universities in China in terms of education system. However, we seldom see care and concern, let alone protection and maintenance. A large number of observations and studies have shown that teachers’ living conditions have seriously deteriorated to the extent that their physical and mental health is at risk. The increasing work and social pressures on teachers at private universities have a significant impact on their job happiness, not only on the stability of the teaching staff at private universities, but also on the quality of teaching and learning, as well as on It also affects the personal development and physical and mental health of teachers, the formation of students’ perceptions and learning outcomes.

Happiness is an important prerequisite for teachers to do a good job in education, a solid foundation for their career success, and an intrinsic motivation for their continuous development (Ding, 2022). In fact, what are the specific factors that affect job happiness of teachers in private universities? And how much does it affect it? It is important to study these issues in order to improve the human resource management of private universities, to improve the teaching ability of teachers and to improve the quality of private higher education (Benevene et al., 2019; Al-Bataineh et al., 2021). The existing literature on quantitative research on factors influencing teachers’ happiness is mainly conducted from the perspectives of job value, compensation and benefits, development prospects, interpersonal relationships, and status differences, with most studies on the relationship between individual factors and job happiness (Jiang, 2008; Chen and Deng, 2009; Lu and Feng, 2011; Sun and Wang, 2012; Li et al., 2014; Wang, 2015). The existing research has two shortcomings, one is that the research on teachers’ job happiness generally adopts a qualitative approach, and most of it comes from scholars’ experience summaries in related work, which is slightly lacking in theoretical height and systematic degree. Secondly, the quantitative analysis of the factors influencing happiness in private universities is mostly on the relationship between individual factors and happiness, and there is little research on the special development environment of private universities, and there is a lack of quantitative analysis of the problem from multiple perspectives and factors, while teachers’ job happiness depends on teachers’ the influence of self and organizational factors. For example, subjective happiness, psychological happiness. Therefore, it is necessary to study the factors influencing happiness based on the existing research and the characteristics of teachers in private universities.

To this end, the present study has found that there are few empirical studies on the happiness of teachers in private universities based on large samples. Thus, this study constructs a questionnaire on the factors influencing teachers’ job happiness and uses multiple linear regression to quantitatively analyze the influence of the factors on teachers’ job happiness in private universities, and then proposes countermeasures to improve teachers’ job happiness in private universities in a targeted manner. This study uses multiple linear regression to quantitatively analyze the influence of the factors on teachers’ job happiness in private universities, and then proposes countermeasures to improve teachers’ job happiness in private universities.

## Theoretical background and hypotheses development

### Theoretical background

Since the 1990s, research on teacher well-being has gradually received attention from international organizations, domestic and foreign scholars, and national governments. After the Organization for economic cooperation and development published “Attracting, Developing and Retaining Quality Teachers” in 2002, some national governments gradually took some measures to enhance teachers’ happiness, and domestic and foreign academics gave more attention to the issue.

The definition of job happiness differs based on the differences in research objectives and criteria. Foreign scholars advocate that job happiness referred to the satisfaction individuals feel at work, such as the realization of personal self-worth and the gain of salary and benefits. Horn et al. (2004) indicated that job happiness is a positive psychological expression of employees in work situations and plays a role in evaluating individual mental health (Page and Vella-Brodrick, 2009). Based on the specific social context of China and in the context of the developmental structure of higher education, studies had shown that the definition of private universities teachers’ job happiness should examine the subjective and psychological experiences of teachers’ practical needs, professional development, and self-worth in their educational work in three dimensions: sense of belonging, sense of growth, and sense of accomplishment (Miao et al., 2009; Wu, 2011; Jia, 2014). The current academic inquiry on job happiness can be summarized into three major types, namely, subjective happiness, psychological happiness and integrated two types of happiness. The main development direction of job happiness research is the mainstream trend of integrated two types of happiness research. The integrated type of job happiness defines job happiness as the overall quality of employees’ experience and effectiveness at work, and the most typical one is the related study by Warr (1990) and Horn et al. (2004). For this reason, the present study uses this as a basis for discussing teachers’ professional happiness.

### Literature review and hypotheses development

#### Professional identity and teachers’ job happiness

Morton and Gray (2010) defined teachers’ sense of professional identity: “Teachers’ sense of professional identity is the emotional experience or psychological feeling that teachers, as individuals and as professionals, fully recognize as a result of various factors, both inside and outside the school and inside and outside the teacher, that influence the work they do as teachers.” Professional identity is a dynamic developmental process of perception, evaluation and repeated interpretation of self-role. Special education teachers’ professional identity has a significant role in influencing their professional happiness. Teachers with higher levels of professional identity have more positive behaviors toward their work, are intrinsically motivated to work hard, and can easily experience the happiness that comes from work (Ren et al., 2021). Now the happiness of teachers in private universities’ corporate establishment is significantly lower than that of teachers in career establishment, and the status of teachers in private universities has a significant effect on their happiness (Wang et al., 2020). Studies have shown that teachers’ professional identity is positively related to well-being (Sun and Wang, 2012). Generally, teachers with low levels of professional identity will evaluate the profession they are in negatively and will view the problems they encounter negatively, which will reduce their professional happiness. On the basis of the analysis of the relationship between the above variables, H1 was formulated in this study.

*H1*: Professional identity is positively related to teachers’ job happiness.

#### Job competence and teachers’ job happiness

Job competence refers to the knowledge, skills, traits, or motivations that are associated with getting the job done or improving job performance. Self-Determine Theory (SDT) is a motivation theory proposed by psychologist Deci, this theory believes that individuals are proactive and naturally pursue development and happiness, and the need for competence is the most basic psychological need to pursue these motivations, and having job competence is an important factor to meet individual happiness (Deci and Ryan, 2000). Waterman’s “Mindstream Theory” also suggests that the improvement of work skills can lead to the individual experiencing self-fulfillment (Waterman et al., 2008). Competent individuals have rich psychological resources such as self-awareness, knowledge, skills, and connections, and these resources enable individuals to take positive actions to cope with various difficulties and meet challenges that arise, and when problems are solved or goals emerge, a benign positive experience is formed, which further enhances their subjective sense of well-being (Stensland and Landsman, 2017). The job competence ability is improved, the work motivation and work efficiency are significantly increased, so that employees can actively establish work goals, find the motivation and direction to make their own efforts, and can enhance the job happiness of employees. On the basis of the analysis of the relationship between the above variables, H2 was formulated in this study.

*H2*: Job competence is positively related to teachers’ job happiness.

#### Professional motivation and teachers’ job happiness

Teachers’ professional motivation refers to an internal psychological state that promotes teachers to engage in education and teaching and thus satisfies their psychological needs.

Self-determination theory is a theory of the motivational process of self-determined human behavior, which divides individual motivation into endogenous, exogenous, and unmotivated (Baard and Deci, 2004). Endogenous motivation refers to teachers’ behaviors that are spontaneous and stem from an interest in their work; exogenous motivation refers to teachers’ efforts to engage in a certain area of work in order to reap certain outcomes. A series of studies have shown that internal motivation can promote positive behaviors and sources of motivation for achievement (Storch et al., 2020). Self-determination theory states that internal motivation not only has an impact on an individual’s behavior and achievement, but also enhances happiness. The professional motivation of teachers in private universities is characterized by diversity, with professional motivation such as interest in teaching, liking to be a teacher, and enjoying the college atmosphere motivating their choice to become teachers, and professional motivation influences the ultimate The professional motivation influences the final job happiness of teachers. On the basis of the analysis of the relationship between the above variables, H3 was formulated in this study.

*H3*: Professional motivation is positively related to teachers’ job happiness.

#### Professional prospects and teachers’ job happiness

Professional prospects refers to the spiritual and material satisfaction that employees can obtain in their career development, and is the desire and aspiration of employees for their careers (Udayar et al., 2021). Professional prospects are influenced by factors such as working conditions, working environment, and working treatment (Anders et al., 2012). As private universities teachers, due to the low social recognition, non-career establishment makes private universities teachers lack of security, academic discrimination and prejudice make private teachers lack of opportunities to improve their teaching and research ability, that as private universities themselves due to the restrictions of funding, resources, and faculty development platform is limited. To a certain extent (Akkermans et al., 2018), this makes private universities teachers feel confused about their own professional prospects, which to a certain extent affects teachers’ enthusiasm and happiness at work. On the basis of the analysis of the relationship between the above variables, H4 was formulated in this study.

*H4*: Professional prospects is positively related to teachers' job happiness.

#### Perceived fairness and teachers’ job happiness

A sense of educational inequity is an important factor affecting happiness, and educational equity itself should be a major component of people’s happiness in life (D’Ambrosio et al., 2018). Fairness in teaching activities directly affects students’ experience of happiness, and students will feel unhappy when faced with unfair situations (Ellorenco et al., 2019). There is a significant relationship between kindergarten teachers’ sense of organizational fairness and professional happiness (Lv and Xie, 2017). Fairness factor is an important factor that affects knowledge workers’ work happiness experience (Ellorenco et al., 2019). According to fairness theory and social identity theory, individuals are more likely to have a comparative mentality within the organization, and through the comparison of results, different perceptions of fairness are generated. Employees who perceive high organizational fairness will identify more with the organization, and are naturally more likely to generate positive emotions and positive behaviors. Fairness in distribution will make knowledge-based employees perceive more deeply the consistency of individual knowledge contribution and reward, as well as the organization’s recognition of individual contribution, resulting in a sense of satisfaction and happiness (Gevrek et al., 2017). In accordance with the above logical derivation, the following hypotheses are proposed in this study.

*H5*: Perceived fairness is positively related to teachers' job happiness.

#### Job achievements and teachers’ job happiness

Teacher job achievements means that teachers believe that they are capable of doing the job they are engaged in, that they have exerted their abilities in the process of education and teaching work (Lavy and Bocker, 2017), which they have fully demonstrated their potential in education and teaching work, which they have achieved their educational and teaching purposes (Baumeister et al., 2003), and that they have reached their pre-planned goals. The study was conducted in a stratified random sample of 353 undergraduates in 4 years from 2 comprehensive universities in Beijing (Manasia and Macovei, 2020). In one study, a stratified random sample of 353 undergraduates in 4 years was selected from two comprehensive universities in Beijing, and the study showed that there was a significant correlation between college students’ achievement motivation and subjective well-being index (Xiyun et al., 2022).

In accordance with the above logical derivation, the following hypotheses are proposed in this study.

*H6*: Job achievements is positively related to teachers' job happiness.

**Figure 1:**
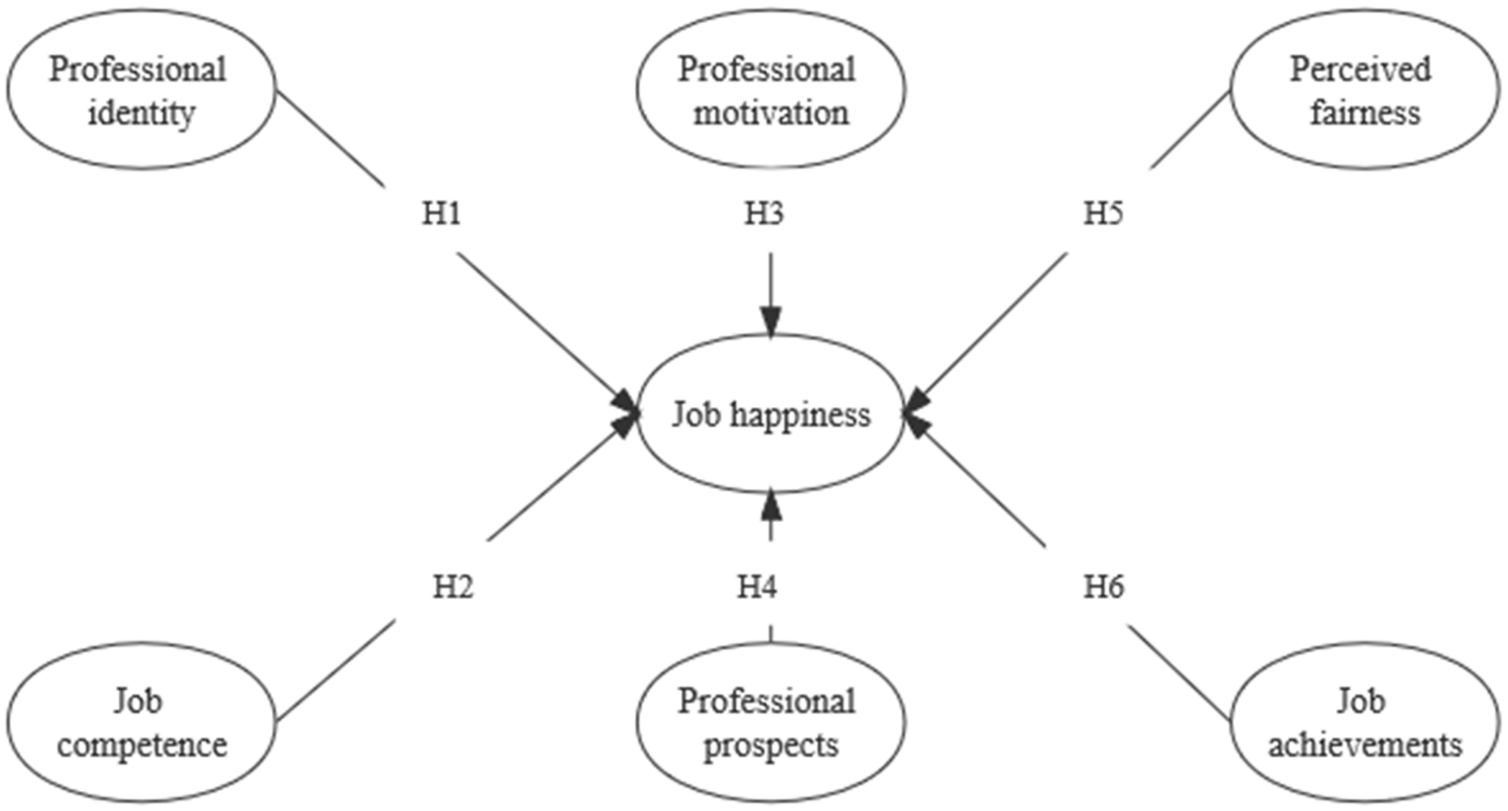
Theoretical model.

According to the above hypothetical relationships, this study constructs the theoretical model as shown in Figure 1.

## Methods

### Participants and procedure

This paper adopts questionnaire research to collect data for empirical study. The survey subjects are mainly teachers of private undergraduate colleges and universities, and the schools are located in the regions of Northeast, Central, East, North, South and West (Northwest and Southwest), involving liberal arts (history, literature, law, philosophy, education, art), science, agriculture and medicine (science, engineering, agriculture, and medicine), business (economics, management), etc. Three major fields. The questionnaire survey of this study lasted for 6 months, starting from May 2021 and ending in November 2021, a total of 2,818 questionnaires were collected, excluding invalid responses, the remaining valid questionnaires totaled 2,181, with a valid return rate of 77.4%, and the statistical analysis of questionnaire data used SPSS24.0 software to count the sample characteristics. The sample specifics are as follows.

The number of women was slightly higher than that of men (68.41%), and the age distribution was dominated by young and middle-aged teachers aged 30–39 (47.46%). In terms of marital status, the largest number of teachers were married (1,577) (72.31%), most teachers were in good health (97.25%), and most of their family members were in good health (95.92%). The largest number of teachers had a master’s degree (54.79%), the majority had the title of lecturer (35.99%), half had 1–5 years of experience (50.62%), and the average monthly income was between RMB 3,001 and RMB 5,000 (47.73%). Half (52.27%) of the full-time faculty were in the liberal arts (history, literature, law, philosophy, education, art). In addition, more than 70% of teachers had part-time jobs, including jobs assigned by their supervisors (77.95%), jobs assigned by the school (72.08%), and part-time jobs outside of teachers’ own working hours (76.71%).

### Measures

Given that private universities teachers’ job happiness is a systemic project that includes the teaching profession itself, the teachers themselves, and the organizational environment of private universities. Therefore, the questionnaire measurement items in this study were derived from the analysis of the literature on the factors influencing teachers’ well-being and were adapted to the actual context. This study constructed seven constructs of professional identity, job competence, professional prospects, perceived fairness, and job achievements, and details of the variable measures and their sources are shown in Table 1. The questionnaires were evaluated using the 5-point Likert scale method except for the basic teacher profile, with 1 indicating “strongly disagree” and 5 indicating “strongly agree.”

**Table 1:** Variable measurements and their sources.

Variable	Item	Sources
Job happiness	I am satisfied with my career as a teacher	Li and Yan (2018); Ding (2022)
	I am satisfied with what I do for a living	
	I find the job of teaching very rewarding	
	I can adapt to every part of the job	
Professional identity	I would be proud to tell others that I am a teacher at this school	Lyngdoh et al. (2018); Li and Yan (2018)
	I feel that my values are similar to the school	
	I stay at the school because it is my responsibility as a part of it to make it better	
	I am willing to go the extra mile to help the school grow	
	I care about the future of the school	
Job competence	I am very confident in my ability to do my job	Sun et al. (2018); Kim et al. (2021)
	I am confident in my ability to do all things at work	
	I have all the skills needed to do my job	
	I can successfully complete the scheduled teaching tasks	
Professional motivation	I chose to be at private universities because I am interested in teaching	Wu et al. (2007); Cai and Dong (2017)
	I chose to be at private universities because I enjoy the process of teaching	
	I chose to work at private universities because I like challenging tasks	
	I chose to work at private universities because I like the work atmosphere at private universities	
Professional prospects	If I continue to stay at our school, there are opportunities to learn	Lin et al. (2012); Zhou (2012); Martins et al. (2014)
	If I stay in our school, I can be promoted	
	If I continue to stay at our school, I have the possibility to achieve my ambition	
	If I continue to stay in our school, it will help me to develop my career	
Perceived fairness	Teachers generally feel that the school has developed a fair reward policy	Kumar et al. (2018); Yang and Wang (2015)
	The school has established some effective rules and regulations to encourage teachers to be innovative	
	Our school has a well-developed teacher development and training mechanism	
	The school has its own strategic goals and teachers are generally willing to work towards them	
	Our school has opportunities for teachers to participate in school governance	
Job achievements	I feel satisfied that I can do a good job in teaching	Deng and Wang (2011); Li and Zheng (2017)
	My teaching performance is recognized and appreciated by my students	
	I am recognized by my colleagues for my teaching performance	
	My teaching performance gives me more confidence	
	My daily teaching work goes well and makes me feel happy	

### Data analysis method

As in the main practice of Structural Equation Modeling (SEM), the collected data were analyzed in this study using Amos 24.0. And the data were analyzed in two parts, measurement model and structural model, as suggested by Bagozzi and Yi (1988).

First, the data analysis method for the measurement model is described as follows. Confirmatory Factor Analysis (CFA) was performed on the data. The convergent validity of all variables were examined by examining the Standardized Factor Loading, Composite Reliability (CR), and Average Variance Extracted (AVE) metrics. Discriminant validity analysis was performed on the data. The convergent validity of all variables were further examined by verifying whether there was a difference between the correlations of two different variables.

Second, the data analysis method of the structural model is described as follows. The model fit was judged according to the recommendations of Jackson et al. (2009), and on this basis, the hypotheses of this study were further tested to provide theoretical support for the final research conclusions and discussions.

## Data analysis and results

### Confirmatory factor analysis

In this study, AMOS 24.0 was used to perform validation factor analysis on the formally administered sample data. According to the recommendations of Anderson and Gerbing (1988), the evaluation of the SEM consists of a measurement model and a structural model. Confirmatory factor analysis (CFA) is equivalent to the estimation of the measurement model in SEM. The measurement model is estimated using the most approximate likelihood estimation method, and the estimated parameters include factor loadings, multivariate correlation square, composite reliability and average variance extracted. Among them, the standardized factor loadings should be greater than 0.60; (2) the Composite Reliability should be greater than 0.60; (3) the Average Variance Extracted should be higher than 0.50; and (4) the Cronbach’a should be higher than 0.50, then the measurement model has good convergent validity (Fornell and Larcker, 1981; Chin, 1998; Hair et al., 2017).

As shown in Table 2, the standardized factor loadings ranged from 0.767–0.925, all of which met the range, showing that each topic had topic reliability; the synthetic reliability of the study constructs ranged from 0.909–0.952, all of which exceeded 0.7, all of which met the criteria suggested by scholars, showing that each construct had good internal consistency; finally, the mean variance extractions ranged from 0.711–0.799, all above 0.5, all meeting the criteria of Fornell and Larcker (1981) and Hair et al. (2017) showing that each construct has good convergent validity.

**Table 2:** Confirmatory factor analysis.

Variable	Item	Factor loadings	Composite reliability	Average variance extracted	Cronbach’a
PI	PI1	0.886	0.952	0.799	0.952
	PI2	0.905			
	PI3	0.903			
	PI4	0.877			
	PI5	0.899			
JC	JC1	0.896	0.935	0.783	0.935
	JC2	0.925			
	JC3	0.850			
	JC4	0.867			
PM	PM1	0.850	0.909	0.715	0.906
	PM2	0.896			
	PM3	0.864			
	PM4	0.767			
PP	PP1	0.840	0.924	0.752	0.924
	PP2	0.798			
	PP3	0.917			
	PP4	0.909			
PF	PF1	0.777	0.925	0.711	0.924
	PF2	0.857			
	PF3	0.876			
	PF4	0.856			
	PF5	0.846			
JA	JA1	0.859	0.937	0.749	0.936
	JA2	0.895			
	JA3	0.893			
	JA4	0.833			
	JA5	0.844			
JH	JH1	0.890	0.935	0.743	0.935
	JH2	0.871			
	JH3	0.863			
	JH4	0.839			
	JH5	0.847			

PI, professional identity; JC, job competence; PM, professional motivation; PP, professional prospects; PF, perceived fairness; JA, job achievements; JH, job happiness.

### Discriminant validity

Fornell and Larcker (1981) suggested that discriminant validity should also take into account the relationship between convergent validity and construct correlation, and therefore suggested that the square root of AVE for each construct must be greater than the correlation coefficient between the constructs, and this condition was met to show that the model in this study had discriminant validity. As shown in Table 3, the root mean square of the AVE of each construct on the diagonal of this study was greater than the off-diagonal correlation coefficient.

**Table 3:** Discriminant validity.

	PI	JC	PM	PP	PF	JA	JH
PI	**0.894**						
JC	0.717	**0.885**					
PM	0.524	0.558	**0.846**				
PP	0.468	0.439	0.736	**0.865**			
PF	0.428	0.385	0.654	0.726	**0.843**		
JA	0.638	0.704	0.583	0.546	0.527	**0.865**	
JH	0.754	0.642	0.550	0.573	0.461	0.584	**0.862**

The items on the diagonal on bold represent the square roots of the AVE; off-diagonal elements are the correlation estimates. PI, professional identity; JC, job competence; PM, professional motivation; PP, professional prospects; PF. perceived fairness; JA. job achievements; JH, job happiness.

It can be seen that the seven dimensional factors of professional identity, job competence, professional motivation, professional prospects, perceived fairness, job achievements, and job happiness have good discriminant validity.

### Structural model analysis

In this study, a method widely used in previous structural equation modeling studies was used to analyze the structural model fit. That is, nine goodness-of-fit metrics were analyzed to determine whether the study model had a good fit (Jackson et al., 2009). In this study, the sample size was greater than 200. Bollen and Stine (1992) stated that if the sample size is greater than 200, it will lead to inflated chi-square values and contribute to a decrease in model fit. Therefore, this study used Bollen-Stine Bootstrap to further correct the SEM cardinality. As corrected using Bollen-Stine Bootstrapping, the model fit indices met all the suggested criteria and all of them were met (see Table 4). Therefore, the structural model in this study has a good model fit.

**Table 4:** Model fit criteria and the test results.

Model fit	Criteria	Model fit of research model
MLχ^2^	The small the better	4496.741
DF	The large the better	443.000
Normed Chi-sqr (χ^2^/DF)	1 < χ^2^/DF < 3	10.151
RMSEA	<0.08	0.065
SRMR	<0.08	0.039
TLI (NNFI)	>0.9	0.935
CFI	>0.9	0.942
GFI	>0.9	0.936
AGFI	>0.9	0.928

### Hypothesis testing

In this research model, the results of the hypothesis testing are shown in Table 5. First, professional identity (PI) (β = 0.226, value of *p* < 0.001) has a positive and significant impact on job happiness (JH). That is, H1 is supported. Second, job competence (JC) (β = 0.264, value of *p* < 0.001) has a positive and significant impact on job happiness (JH). That is, H2 is supported. Third, professional motivation (PM) (β = 0.287, value of *p* < 0.001) has a positive and significant impact on job happiness (JH). That is, H3 is supported. Fourth, professional prospects (PP) (β = 0.275, value of *p* < 0.001) has a positive and significant impact on job happiness (JH). That is, H4 is supported. Fifth, perceived fairness (PF) (β = 0.261, value of *p* < 0.001) has a positive and significant impact on job happiness (JH). That is, H5 is supported. Sixth, job achievements (JA) (β = 0.218, value of *p* < 0.001) has a positive and significant impact on job happiness (JH). That is, H6 is supported.

**Table 5:** Regression coefficient.

Hypothesis	Unstd. coefficient (β)	SE	*z*-Value	Std. coefficient	Value of *p*	R-square	Result
PI- > JH	0.326	0.051	4.431	0.367	***	0.311	Supported
JC- > JH	0.264	0.057	4.561	0.340	*	0.315	Supported
PM- > JH	0.287	0.061	4.705	0.328	***	0.512	Supported
PP- > JH	0.275	0.068	4.561	0.243	**	0.367	Supported
PF- > JH	0.261	0.057	4.579	0.274	***	0.622	Supported
JA- > JH	0.218	0.041	5.317	0.271	***	0.546	Supported

* *p* < 0.05;

** *p* < 0.01;

*** *p* < 0.001.

PI, professional identity; JC, job competence; PM, professional motivation; PP, professional prospects; PF, perceived fairness; JA, job achievements; JH, job happiness.

The explanatory power of professional identity to explain job happiness is 31.1%. The explanatory power of job competence to explain job happiness is 31.5%. The explanatory power of professional motivation to explain job happiness is 51.2%. The explanatory power of professional prospects to explain job happiness is 36.7%. The explanatory power of perceived fairness to explain job happiness is 62.2%. The explanatory power of job achievements to explain job happiness is 54.6%. This indicates that the research model has good explanatory power.

## Discussion

The results of data analysis show that among the factors influencing private universities teachers’ job happiness, perceived fairness, professional identity, job achievements, job competence, professional professional prospects, and professional motivation have significant positive effects on private universities teachers’ job happiness, in descending order of influence, professional identity, job competence, professional prospects, and professional motivation have significant positive effects on private universities teachers’ job happiness. Professional prospects, perceived fairness, job achievements, and professional motivation. The details are discussed as follows.

First, the results of data analysis showed that professional identity has a significant effect on job happiness. The findings are consistent with the logical reasoning of the hypothesis and professional identity has the greatest effect on private universities teachers’ job happiness. The results of the hypothesis test showed that professional identity was significantly and positively related to private universities teachers’ job happiness, with the largest unstandardized coefficients (β) of 0.326, which was the largest among the six factors. That is, any small improvement in teachers’ professional identity will lead to an increase in teachers’ job happiness in private universities. Teachers with a strong sense of professional identity in private universities are proud to be teachers and are driven by a strong sense of self-development, which allows teachers to develop an intrinsic interest in teaching and to find enjoyment and fulfillment in it, which is the prerequisite foundation for teachers to strive to do their jobs well and to continue to forge ahead. Only if they love the teaching profession enough can teachers consciously and voluntarily achieve self-development, treating the process of pursuing continuous improvement of teaching ability as an internal positive development process, pursuing life values, and not catering to external pressure or temptation, in order to continuously improve the quality of teaching and talent training.

Second, the results of data analysis showed that job competence has a significant effect on job happiness. The findings are consistent with the logical reasoning of the hypothesis and the effect of job competence on private universities teachers’ job happiness is second to none. Job competence has a significant positive effect on private universities teachers’ job happiness, with a standardized regression coefficient second only to professional identity. Teachers of private universities need to keep up with the times, learn and enrich themselves, have the ability to apply modern teaching techniques, organize and design teaching, and know how to translate subject knowledge into subject teaching. It is also important for teachers to be able to stimulate the interest in learning according to the characteristics of the student population of private universities, so that the course can be “knowledgeable + interesting + practical” and improve the effectiveness of the class. This is easy to be recognized by the leaders and admired by the students, which is conducive to maintaining a good working condition, thus realizing self-worth and reaping the happiness of teachers’ work.

Third, the results of data analysis showed that professional prospects had a significant effect on job happiness. The findings are consistent with the logical reasoning of the hypothesis. The unstandardized coefficients (β) of professional prospects on private universities teachers’ job happiness was 0.275. This indicates that the more promising private universities teachers’ careers are, the more they feel happy at work. If teachers feel that in private universities, there are clear promotion channels, room for development, a specialized teacher training system, and many learning opportunities, they will feel that being a teacher in a university is helpful for their future career development, which can also enhance teachers’ job happiness.

Fourth, the results of data analysis showed that perceived fairness had a significant effect on job happiness. The findings are consistent with the logical reasoning of the hypothesis. The development of teachers in private universities is a long-term process, in which the establishment of appropriate management systems is the basic guarantee of teacher development. Private universities’ management systems are not perfect, management systems are changed from time to time, systems are not effectively implemented, performance assessment mechanisms, training mechanisms are not perfect, etc., which shackle the development of teachers in private universities is still in existence. In practice, only teachers feel that the school’s performance appraisal mechanism is fair, the school provides sufficient training and development opportunities for teachers, the school’s various management mechanisms are more standardized and reflect a people-oriented approach, teachers can feel fair in such an organizational environment, and working in such an atmosphere will greatly enhance job happiness.

Fifth, the results of data analysis showed that job achievements had a significant effect on job happiness. The findings are consistent with the logical reasoning of the hypothesis. Teachers’ strong job achievements motivate them to devote themselves to their work, to obtain achievements, and to generate a sense of accomplishment, pleasure, and responsibility. Work is no longer passive, but becomes a conscious and autonomous behavior. Teachers need to have a sense of accomplishment in teaching. It is a great honor and pleasure to be recognized and sought after by students through successfully teaching each lesson, and to feel the value and pleasure of teaching from it. Teachers are not only teaching, but also influencing their students to develop into the talents needed by society through their own words and example. This is the greatest sense of accomplishment as a teacher. Teaching is a profession that requires selfless dedication, especially for teachers in private universities, where the quality of students is weak and the salary and benefits in private universities are not as good as in public schools, teachers need to have enough sense of job achievements in order to enhance their The teachers need to have enough sense of job achievements in order to enhance the teachers’ happiness in their work.

Sixth, the results of data analysis showed that professional motivation had a significant effect on job happiness. The findings are consistent with the logical reasoning of the hypothesis. The unstandardized coefficients (β) of professional motivation on private universities teachers’ job happiness is 0.287. private universities are influenced by funding and resources, and social recognition is low, and since private The operation of private universities is subject to cost control, and they mainly rely on tuition fees to maintain their survival and development, and there is a gap between teachers’ welfare and public universities. In such a difficult situation, teachers of private universities need to have educational emotions, enjoy the teaching process, truly love education, be interested in teaching, and reap a sense of accomplishment from the growth of students in the process of education and teaching.

They should have clear and simple professional motivation, strong educational feelings, deep love for the teaching profession, and the ability to quietly enjoy the happiness brought by this profession without being influenced by the external environment.

## Practical implications

This study is a quantitative study of the factors influencing teachers’ job happiness in private universities. The model of private universities teachers’ job happiness influencing factors constructed in this study provides theoretical support for the in-depth understanding and effective prediction of private universities teachers’ job happiness, and has implications for private universities. This study provides theoretical support for understanding and effectively predicting teachers’ job happiness in private universities.

Firstly, the government should pay attention to the internal development of private higher education and improve the professional identity of teachers. For a long time, the state has focused on the compliance of private universities’ development, and has provided little support to private universities’ financial investment, teachers’ salary and humanistic care, talent training and scientific research. The lack of resources is an important factor affecting the change of the faculty development system of private universities, and it is also a bottleneck restricting the improvement of teaching ability level of private higher education teachers. The policy for teachers in private universities should be changed from simple and traditional management to value-based management for their development. To improve the initiative of private universities and teachers, we need to promote the change of faculty development system. We should increase the financial support for faculty development in private universities, and support the establishment of faculty development centers in private universities in terms of resources and policies; encourage universities to use specific faculty development programs as the basis, make full use of social resources to improve teachers’ teaching ability, and expand the funding sources for faculty development according to their own characteristics In addition, we also encourage universities to make full use of the support of social resources to enhance teachers’ teaching ability and expand the funding sources for teacher development. Private universities once made a significant contribution to the popularization of higher education in China, and in the new era, the high-quality development of higher education in China is inseparable from private higher education, as private universities ushered in the classification and management of non-profit private universities, the state needs to introduce some policies and measures to promote the Private universities need to introduce policies and measures to promote the high-quality development of private universities, teachers are the main body of the high-quality development of private universities, so that private universities teachers can have the same development opportunities as teachers in public universities, enhance the social recognition of private universities teachers This will help to improve the professional identity of private universities teachers and greatly enhance the job happiness of private universities teachers.

Secondly, private universities should strengthen their organizational support for faculty development. On the one hand, it is important to establish a regular mechanism to promote teachers’ teaching ability and improve job competence. Science and technology are changing rapidly, and schools help teachers adapt to and face the challenges of new ideas, new knowledge and new technology, so that teachers’ job skills can keep up with the requirements of high-quality development of higher education, and schools should have different levels and types of training systems for teachers’ teaching competence improvement. Teachers with different needs can find the right training content to learn. The university actively builds a platform for scientific research, improves the scientific research management system, establishes, and cultivates an atmosphere for teachers to engage in scientific research, improves scientific research facilities, research materials, flexible systems and mechanisms, advocates scientific research integrity, improves teachers’ scientific research literacy, and organizes teachers to actively declare vertical and horizontal projects. As teachers, they must pay attention to the positive correlation between scientific research and teaching, carry out research around teaching, and give full play to the role of scientific research in promoting teaching. This will help deepen teaching content, provide richer knowledge content for students’ learning, improve students’ learning effect, gain students’ and parents’ recognition, harvest job achievements and enhance job happiness.

On the other hand, it is important to guide teachers to establish that devoting oneself to education is an important way to realize the value of life. There may be many ways to realize life value, especially in the diverse life paths choose teaching career may not bring more material wealth to individuals, but the teaching profession can meet the conditions of basic material life to train talents for society and dedicate to scientific career, and then can obtain spiritual wealth. In our higher education system, private universities have a high level of stress and heavy workload, which is the consensus of the higher education industry. This is a common understanding of the higher education industry. It is common in the higher education industry that teachers should be guided to adapt to the working environment of private universities, and to carry out activities to improve teachers’ resistance to stress and to adapt to the working atmosphere of private universities. Meanwhile, private universities should take the initiative to improve their internal management and create a harmonious environment for their work, so that teachers can fall in love with the teaching profession, learn for life, and improve their teaching skills.

Thirdly, to build a developmental evaluation mechanism for teachers of private universities. Teacher evaluation mechanism is a basic measure for scientific and efficient management and effective motivation of university faculty. The main purpose of building an effective evaluation mechanism is to make teachers happier in school, to enhance teachers’ job happiness, and to realize the development of teachers’ teaching ability. Construct a developmental teacher evaluation system. The traditional teacher evaluation used to focus more on teachers’ past qualities and achievements, and lacked the function of motivating teachers to improve, which made it difficult to promote the development of teachers’ teaching ability. Therefore, it is necessary to establish an up-to-date evaluation system to make teachers perceive fairness, to encourage them to develop their teaching abilities, to encourage them to teach according to their abilities, to require them to reflect on their teaching, and to make them proactively respond to the needs of higher education in the age of digitalization.

This study uses quantitative research methods to analyze the influencing factors of private universities teachers’ job happiness, and then proposes countermeasures to improve private universities teachers’ job happiness with a strong theoretical basis and certain systematicity, which can promote the development and improvement of private universities’ teachers’ team construction.

## Theoretical contributions

This study explores the specific factors influencing private universities teachers’ job happiness based on empirical research, and the theoretical contributions are mainly as follows.

First, this study explores the factors influencing private universities teachers’ job happiness in an all-round way by taking private universities teachers as the research target, and expands the related research. Using research data from six regions: northeast, central, east, north, south, and west (northwest and southwest), we conducted an empirical study on the happiness of teachers in private universities through a large sample, and achieved a comprehensive analysis of factors affecting the happiness of teachers in private universities from a multi-angle perspective for the special development environment of private universities. The study also constructs the theoretical relationship between professional identity, job competence, professional motivation, professional prospects, perceived fairness, and job happiness. The theoretical model of the relationship between professional identity, job competence, professional motivation, professional prospects, perceived fairness, job happiness, and job achievements.

Second, this study adopts a quantitative research method to provide an in-depth analysis of the factors influencing teachers’ job happiness in private universities in China and to provide specific paths for improving human resource management in private universities. The results of quantitative analysis show that professional identity, job competence, professional prospects, perceived fairness, job achievements, and professional motivation all have significant effects on teachers’ job happiness in private universities, so as to clarify the influence of each factor on job happiness and provide a specific path for private It is also important to clarify the influence of each factor on job happiness, to provide a specific path for private universities to improve teachers’ job happiness, to optimize the level of human resource management in private universities, and to improve the organizational performance of private universities.

## Research limitations and future research directions

The context of this study is domestic universities, and the research method also only uses quantitative research methods, using questionnaires to collect questionnaires. Due to the constraints of time, energy, economic conditions, and many other factors, in-depth interviews were not conducted; the geographical distribution of the selected questionnaire sample involved six regions though. However, the sample size of each region was not very large. This may also affect the generalizability of the results of this study. In addition, although this study has verified the influencing factors of job happiness, it has not explored the mechanism of each influencing factor on job happiness in more depth, such as through what internal mechanism professional identity affects teachers’ job happiness, whether there is an interactive relationship between job The study has not explored more deeply the mechanism of the influence of various factors on job happiness, such as how professional identity affects teachers’ job happiness, whether there is an interactive relationship between factors such as competence and professional prospects. In response to the above problems, the sample coverage and sample size can be expanded in the future to better investigate the teachers of private universities distributed in different regions, to improve the representativeness and universality of the research sample, and to facilitate in-depth data mining and generalization. In the future, we can also conduct a study on the critical path of teachers’ job happiness and propose a more effective and reasonable plan for the construction of teachers’ management team in private universities.

## Data availability statement

The original contributions presented in the study are included in the article/supplementary material, further inquiries can be directed to the corresponding author.

## Ethics statement

Ethical review and approval were not required for the study on human participants in accordance with the local legislation and institutional requirements. Written informed consent from the participants was not required to participate in this study in accordance with the national legislation and the institutional requirements.

## Author contributions

BC: conceptualization. BC and GR: formal analysis. BC and YL: investigation. BC, GR, and YL: writing original draft and writing – review and editing. All authors have read and agreed to the published version of the manuscript.

## Funding

This study was supported by the humanities and social sciences research planning fund project of the Ministry of Education of the People’s Republic of China, “Research on the Formation, Evaluation and Improvement of Production and Education Integration Ability of Teachers in Applied Universities” (21YJA880037).

## Conflict of interest

The authors declare that the research was conducted in the absence of any commercial or financial relationships that could be construed as a potential conflict of interest.

## Publisher’s note

All claims expressed in this article are solely those of the authors and do not necessarily represent those of their affiliated organizations, or those of the publisher, the editors and the reviewers. Any product that may be evaluated in this article, or claim that may be made by its manufacturer, is not guaranteed or endorsed by the publisher.
